# Avian Paramyxovirus Serotype-1: A Review of Disease Distribution, Clinical Symptoms, and Laboratory Diagnostics

**DOI:** 10.1155/2012/708216

**Published:** 2012-04-11

**Authors:** Nichole L. Hines, Cathy L. Miller

**Affiliations:** ^1^National Veterinary Services Laboratories, Animal and Plant Health Inspection Service, United States Department of Agriculture, Ames, IA 50010, USA; ^2^College of Veterinary Medicine, Iowa State University, VMRI Building 3, Ames, IA 50011, USA

## Abstract

Avian paramyxovirus serotype-1 (APMV-1) is capable of infecting a wide range of avian species leading to a broad range of clinical symptoms. Ease of transmission has allowed the virus to spread worldwide with varying degrees of virulence depending on the virus strain and host species. Classification systems have been designed to group isolates based on their genetic composition. The genetic composition of the fusion gene cleavage site plays an important role in virulence. Presence of multiple basic amino acids at the cleavage site allows enzymatic cleavage of the fusion protein enabling virulent viruses to spread systemically. Diagnostic tests, including virus isolation, real-time reverse-transcription PCR, and sequencing, are used to characterize the virus and identify virulent strains. Genetic diversity within APMV-1 demonstrates the need for continual monitoring for changes that may arise requiring modifications to the molecular assays to maintain their usefulness for diagnostic testing.

## 1. Introduction

Avian paramyxovirus serotype-1 (APMV-1) is a member of the Paramyxoviridae family and is the causative agent of virulent Newcastle disease (vND). The virus is able to infect all orders of avian species, and virulent strains can cause significant clinical signs. Due to the extensive range of susceptible hosts, the virus has been able to establish itself worldwide. Infection by virulent strains has resulted in several panzootics since 1926 [1–3]. This disease can have devastating effects on the poultry industry due to the high morbidity and mortality associated with virulent strains of the virus [4–6]. Clinical signs of vND include drop in egg production, respiratory distress, listlessness, weakness, and central nervous system symptoms [2]. Vaccination programs exist within the United States (US), but the virus continues to replicate upon infection and can spread from infected vaccinated flocks. Currently the US is free of vND, but introduction of the disease continues to be a major concern for the agricultural community [2].

 Illegal importation of infected birds is one of the major modes of vND introduction into the US. Diagnostic testing and rapid detection are important steps to prevent an outbreak of the disease. Real-time reverse transcription polymerase chain reaction (rRT-PCR) is a rapid diagnostic test for detection of APMV-1 RNA. Virus isolation in embryonating chicken eggs is the “gold standard” method of virus identification but can require 5 to 10 days to obtain an isolate. The current United States Department of Agriculture- (USDA-) validated rRT-PCR assay used at the National Veterinary Services Laboratories (NVSL) is designed to detect the matrix gene of most strains of APMV-1 [4, 7–10]. Studies have shown that some strains of APMV-1 such as lineage 6 (Class I) and some pigeon paramyxoviruses (PPMV-1) are not detected by the primer/probe set used in this assay [7, 8].

 The matrix rRT-PCR assay is able to detect APMV-1 RNA within 3 hours of sample receipt in the laboratory. The NVSL uses this as an important screening assay allowing for a quick turn-around time for reporting results. Lack of detection by the matrix assay can result in a 7-to-14-day delay in reporting detection of the virus. Development of an rRT-PCR assay that can detect a broad range of APMV-1 will increase the diagnostic capability of the NVSL and the laboratories of the National Animal Health Laboratory Network (NAHLN) in the US.

 When APMV-1 RNA is detected by the matrix rRT-PCR assay, additional testing is required for that specimen to determine if the RNA originated from a virulent strain. The USDA-validated fusion gene rRT-PCR assay is a pathotyping assay used to detect strains of vNDV. This assay allows for rapid identification of vNDV also within 3 hours of sample receipt. The fusion gene rRT-PCR assay used at the NVSL and the NAHLN laboratories is also limited in the strains of vNDV that it is able to detect. Cormorant vNDV and most strains of PPMV-1 are not detected using this fusion gene assay [7, 11]. Although cormorant vNDV and PPMV-1 are not highly infective to poultry, rapid detection is still important in diagnosing APMV-1 infection. Development of an rRT-PCR assay specific for these strains would allow laboratories to easily distinguish cormorant vNDV or PPMV-1 from strains of vNDV that are highly contagious to poultry.

## 2. Classification

There are 9 serotypes of avian paramyxovirus (APMV-1 to APMV-9) capable of infecting avian species [2, 12–14]. Newcastle disease virus (NDV) falls into the avian paramyxovirus serotype 1 (APMV-1). APMV-1 is a member of the order mononegavirales in the Family Paramyxoviridae [2, 12, 15, 16]. This family is broken down into two subfamilies the Paramyxovirinae and the Pneumovirinae. The paramyxovirus family includes many significant human and animal pathogens that cause severe disease such as measles, mumps, Hendra, Nipah, human respiratory syncytial virus, human parainfluenza viruses 1–4, parainfluenza virus 5, Sendai virus, and NDV infections. Rearrangement of the Paramyxoviridae family by the International Committee on the Taxonomy of Viruses in 1993 placed APMV-1 in the *Rubulavirus* genus. Since that time differences among the Paramyxoviridae family lead to development of a new *Avulavirus* genus.

 The Paramyxovirinae and Pneumovirinae subfamilies differ by several distinct characteristics. Morphologically, pneumoviruses have narrower nucleocapsids and the genome encodes more proteins than paramyxoviruses [15–18]. Pneumoviruses encode a unique SH protein which is expressed as a type II integral membrane protein [15, 16]. The protein locates to the plasma membrane and becomes packaged as part of the envelope upon release of progeny virion. These viruses also encode accessory proteins NS1 and NS2 along with two M2 matrix proteins which differ from the matrix protein of paramyxoviruses. Antigenic sites of paramyxoviruses are capable of cross-reacting and are unique from antigenic sites found on the surface of pneumoviruses [17, 18]. The hemagglutinin-neuraminidase (HN) surface glycoprotein protein of paramyxoviruses is capable of both hemagglutination and neuraminidase activities, while the surface glycoprotein (G) of pneumoviruses does not have the neuraminidase function.

 APMV-1 has a gene map structure similar to rubulaviruses which is the reason for the initial classification [15, 16]. Further analysis discovered that unlike other rubulaviruses, APMV-1 lacks a C protein, a small hydrophobic (SH) protein and the phosphoprotein (P) is relatively small [15, 16, 19]. The intergenic region is also variable compared to other Rubulaviruses. Some features such as nucleotide sequence identity at the conserved genomic termini and RNA editing make it appear similar to Respiroviruses. The nucleotide sequence does not align with Rubulaviruses nor Respiroviruses leading to the new classification under the *Avulavirus* genus.

 Two different classification schemes for NDV are used to group isolates based on genetic analysis [6, 8, 14, 20, 21]. Differences in groupings arise between the two classification methods and either can be used based on preference. One classification proposed by Aldous et al. is based on genotypes or genetic lineages grouped under serotype 1 (APMV-1) [21]. This grouping scheme divides NDV into six lineages (lineages 1 to 6) [12]. Sublineages (a to d) were created in lineages 3 and 4, while sublineages (a to e) were formed in lineage 5. These genetic groupings are indicated by lineage and sublineage such as 3a and 3b. A second classification method based on the genomic characterization and sequence analysis of the F and L genes groups isolates into either Class I or Class II as opposed to lineages [4, 6, 8, 20, 22, 23]. Isolates from Class I are present in the US Live Bird Markets, domestic poultry, and wild waterfowl. Class I is composed of primarily low virulent isolates, but one virulent isolate has been included in that classification. Class I viruses have a worldwide distribution and are further divided into nine genotypes. Isolates grouped in Class I have the longest APMV-1 genome at 15,198 nucleotides. Class I isolates are not usually reported to OIE due to their low virulence designation.

Isolates causing all four panzootics from 1920 to the present are classified as Class II [20]. Class II viruses are usually recovered from poultry, pet birds, and wild waterfowl. Class II viruses are further divided into genotypes I through IX. Genotypes I through IV and IX have slightly shorter genome lengths at 15,186 nucleotides. These genotypes are considered “early” due to their identification between 1930 and 1960. Genotypes V through VIII and X have a medium length genome at 15,192 and are considered “late” due to their identification after 1960. All vNDV are classified as Class II except for one isolate which caused the Australian outbreak from 1998 to 2000. This isolate was determined to originate from a low virulent strain of NDV (LoNDV) which increased in pathogenicity after circulating through poultry [4, 6, 20]. This may explain the classification in Class I where all other isolates are LoNDV.

LaSota, B1, and Villegas-Glisson/University of Georgia (VG/GA) vaccine virus strains are classified as Class II, genotype II [6, 23]. The velogenic neurotropic NDV (vnNDV) Chicken/Texas GB/1948 is also a member of genotype II indicating the broad range of isolates which can be assigned to one genotype. Genotypes V through VIII consist of only vNDV isolates and have a worldwide distribution. The 1971 and 2002 California and 1971 and 1993 Florida outbreaks were caused by genotype V [6, 11, 23]. Cormorant vNDV is classified under genotype V, while PPMV-1 is classified under subgenotype VIb. All other Class II genotypes include viruses isolated from outside of the US.

## 3. Discovery of Avian Paramyxovirus Serotype-1

The virulent form of NDV was first discovered in Java, Indonesia, and Newcastle upon Tyne region in England in 1926 [1–3, 20, 24]. Historical data indicate that outbreaks in poultry with symptoms similar to those seen with vND may have been present in Korea prior to 1926 and also in Scotland as early as 1896. According to Hanson, there are three hypotheses to explain the sudden occurrence of vND in Southeast Asia [25]. First, it is possible that vND was endemic in Southeast Asia and only became a problem when poultry became commercialized [1, 25]. The second theory is that vND was present in bird species living in the tropical rain forest and was introduced into poultry by man similar to the way the movement of tropical birds spread the disease today. The third explanation is that a major mutation occurred in the precursor virus allowing for a change in pathogenicity from low virulence to high virulence. Having the ability to infect all orders of avian species, APMV-1 has been able to spread throughout the world resulting in four panzootics [1, 4, 14, 26–28]. The initial panzootic took 20 years to develop spreading very slowly throughout the world [1]. The United States (US) was likely not involved in the first panzootic but was not so lucky during the second panzootic. The second outbreak spread at a much faster rate, taking only 4 years to spread throughout the world. Globalization and the development of various modes of transportation led to the increased rate of disease spread during the second, third, and fourth panzootics occurring in 1960, the late 1970s, and the 1980s, respectively.

 The term “Newcastle disease” was coined by Doyle as a temporary name to distinguish it from other diseases at the time [1, 2]. The name was never changed, but APMV-1 has become an alternative term used interchangeably with NDV [4, 8]. Despite being a synonym for APMV-1, the term “NDV” has recently evolved to describe the more virulent forms of the disease while APMV-1 encompasses all strains of serotype 1 including asymptomatic, low virulent, and highly virulent strains. Substantial evolution of APMV-1 led to the formation of a separate clade of virulent NDV discovered in 2003 [11]. A virulent strain of NDV emerged between 1995 and 2000 affecting Double-Crested Cormorants in Canada. This strain causes significant mortality in juvenile cormorants and poses a risk to other avian species including poultry.

 Pigeon paramyxovirus-1 (PPMV-1) is another strain of APMV-1 which originated in pigeons [1, 3, 26]. This variant of NDV was discovered in the Middle East during the third panzootic in the 1970s. Like the NDV mentioned previously, this disease has spread easily and now has a global distribution. In 1984 the virus spread from pigeons into domestic poultry in Great Britain. Contamination of feedstuffs with pigeon feces led to 23 outbreaks in commercial chickens. These outbreaks indicated the virus had the ability to replicate and cause infection in other avian species. The disease was no longer limited to feral pigeons and could be a source of economic loss. PPMV-1 disease in pigeons has been an ongoing panzootic since the 1980s [1, 2]. It remains an endemic disease in several countries due to lack of vaccination, housing methods, and the sport of pigeon racing.

## 4. World Distribution

The World Organization for Animal Health (Office International des Epizooties, OIE) defines reportable NDV as an APMV-1 infection in birds which meets the following criteria for determining virulence: the intracerebral pathogenicity index (ICPI) in day-old chicks is greater than or equal to 0.7 or the carboxyl (C-) terminus of the F_2_ protein contains multiple basic amino acids and phenylalanine at residue 117 of the F_1_ protein N-terminus [1–3, 7, 29, 30]. The presence of at least three lysine or arginine residues between positions 113 and 116 defines the term “multiple basic amino acids.” It is difficult to track the geographic distribution of vND throughout the world due to limited reporting to the OIE. Some countries only report when the disease is present in commercial poultry and not when it erupts in backyard flocks. Use of live vaccines can also interfere with the ability to distinguish current infections with vaccinations in part due to the variety of strains used in live virus vaccines.

 Epizootics continue to occur on a regular basis in Central and South America, Africa, and Asia, while sporadic epizootics occur in Europe [1]. An increase in outbreaks in Western Europe began in the 1990s. Several strains were shown to be responsible for these outbreaks through phylogenetic and antigenic evaluations. Backyard poultry continues to be commonly infected in European countries including outbreaks in 1991–1995 and 2000. The following outbreaks occurred between 1995 and 1999: 18 in Denmark, 27 in Northern Ireland, two in Finland, one in Sweden, one in the Republic of Ireland, and one in Norway. Several outbreaks have occurred in Australia including one in 1932, 1998, 1999 and 2000. Of these outbreaks, the ones in the Republic of Ireland (1990) and in Australia (1998–2000) were shown to originate from an increase in virulence from low virulent ND to vND after replication in poultry [4, 6, 20]. In 2008 outbreaks were reported in the Dominican Republic, Belize, Peru, Finland, Germany, and Japan [6]. Despite lack of reporting to the OIE, NDV remains an endemic disease in parts of Africa and Asia.

## 5. Outbreaks in the US

A disease termed “pneumoencephalitis,” serologically indistinguishable from APMV-1, was discovered in the US in the 1930s [1, 2, 23]. As panzootics of the disease occurred, the importation of caged and exotic birds into California caused outbreaks in the 1970's [1–3, 31]. Regulation of importing birds has become strict, reducing the occurrence of the disease in the US. Despite importation quarantine procedures, exotic birds are still smuggled into the country on a regular basis. Virulent NDV is often isolated from illegally imported and quarantined birds. In 1991 six states were affected by vND from illegally imported pet birds [2]. Fortunately the disease was not transmitted to poultry during that outbreak.

The practice of fighting cocks has also lead to the introduction of vND into the US [2]. In 1975, 1998 and 2002-2003 game fowl were to blame for three separate outbreaks of vND. The 2002-2003 outbreak in California caused the most significant economic loss resulting in the depopulation of more than 3 million birds on 2,671 premises including 21 commercial table-egg layer flocks [2, 23, 32]. Transportation of infected birds or contaminated material and transmissibility of the disease led to subsequent outbreaks in Nevada, Arizona, and Texas. Efforts to eradicate the disease cost the US an estimated $180 to $360 million. Coordinated eradication efforts helped to end the outbreak by 2003. Since that time, the US has been free of vND in poultry [4].

 Currently low virulent strains of APMV-1 are endemic in the US [4, 23]. A majority of the field isolates are lentogenic, but virulent strains of NDV cause outbreaks in double-crested cormorants [2, 3, 11]. Although adult cormorants are considered to be the natural reservoir for this strain of vNDV, juvenile cormorants are highly susceptible to the disease. In 1990 and 1992 cormorant vND caused mortality events in double-crested cormorants and pelicans. Isolates from epidemics in the north central US and southern California were classified as velogenic neurotropic viruses, meaning these virulent strains caused clinical disease of the nervous system [3]. Outbreaks have also occurred as recently as 2008 and 2010 [33]. These vNDV strains are usually restricted to cormorants, but in 1992 an outbreak of cormorant vND occurred in turkeys in North Dakota [2]. Reoccurrence of the disease in poultry has not been seen since that time. Cormorant vNDV is reportable to the OIE due to the high ICPI values and presence of multiple basic amino acids at the fusion gene cleavage site [11]. All other virulent strains of NDV are considered exotic to the US leading to the term “exotic Newcastle disease” (END) [6, 23, 27]. The California outbreak in 2002 was one example of the widespread use of the term “END.”

PPMV-1 was first introduced into the US at the same time as the Great Britain outbreak in 1984 [3, 26]. In the year following the initial isolation in New York, the NVSL collected 34 additional isolates of PPMV-1 primarily from the eastern US. The virus spread throughout the US. and has been isolated from feral and domestic pigeons since that time. Texas and Georgia experienced a severe form of the disease in 1998, leading to concerns of an introduction into commercial chickens similar to the outbreaks in Great Britain. Up to this point, natural transmission of PPMV-1 to domestic chickens has not occurred in the US. [3]. PPMV-1 continues to be endemic in feral and racing pigeons in the US, and doves have also been shown to harbor the disease. Pigeons have become the natural reservoir for PPMV-1 [11].

The Agricultural Bioterrorism Protection Act of 2002 established strict guidelines and procedures to control the possession, use, and transfer of biological agents that pose a threat to animal health [32, 34, 35]. All virulent strains of Newcastle disease are List A biological agents classified as Select Agents under the Code of Federal Regulations [6, 19, 32, 34]. Strict handling procedure must be followed when working with this agent. Isolation or acquisition of vNDV must be immediately reported to the Animal and Plant Health Inspection Service (APHIS) and/or the Centers for Disease Control and Prevention (CDC). In order to work with vNDV in the US, a facility must be registered with either the APHIS or CDC. Isolation of vNDV is reportable to the OIE and can lead to international trade restrictions; therefore, disease-free status is needed to maintain poultry exports from the US [1, 3, 6, 8, 24].

## 6. Viral Proteins

APMV-1 is an enveloped, pleomorphic, nonsegmented, negative-sense, single-stranded RNA virus which is approximately 15.2 kb [2, 3, 15, 16, 36]. The genome encodes six proteins including the nucleocapsid (NP), phosphoprotein (P), matrix (M), fusion (F), hemagglutinin-neuraminidase (HN), and the RNA-dependent RNA polymerase (L). The virion is composed of a stable nucleocapsid core consisting of the NP protein bound to the genomic and antigenomic RNA [14–16, 37, 38]. The P and L proteins bind to the nucleocapsid core shortly after synthesis to form the ribonucleoprotein (RNP) complex. This RNP complex becomes the template for transcription by the RNA-dependent RNA polymerase L protein. The L protein binds the genomic RNA at a 3′ entry site in the RNP complex and transcribes the six protein genes using a start-stop mechanism. In this mechanism the L protein initiates transcription and releases the RNP complex after transcribing a number of nucleotides along the gene which for the Paramyxoviridae family is always equal to some multiple of six nucleotides. This transcription requirement is referred to as the “rule of six” [14, 37].

Transcription creates a gradient of messenger RNA (mRNA) protein transcripts in order from the 3′-NP-P-M-F-HN-L-5′. Protein gene proximity to the 3′ end results in a higher production of the protein. The Paramyxovirinae subfamily requires the genome length to be a multiple of six nucleotides for efficient replication. The NP subunit must be in contact with six nucleotides at a time which is termed the “rule of six” [14, 37]. A shift in the NP subunit on the genomic or antigenomic RNA results in a shift in the promoter position leading to incorrect or inefficient replication.

The ND virion contains two types of surface glycoproteins, the F protein and the HN protein [2, 15, 16, 38–43]. The F protein is a class I fusion glycoprotein which is synthesized as a type I integral membrane protein. When the protein is translated, three identical polypeptide chains assemble into homotrimers. Carbohydrate chains are posttranslationally added to the homotrimers which are biologically inactive. Host proteases must cleave the precursor protein in order for it to become biologically active.

The F protein cleavage site of velogenic and mesogenic strains (including PPMV-1 and cormorant vNDV) contains a furin recognition site with multiple basic amino acids (arginine or lysine) surrounding the glutamine at position 114 (C-terminus of F_2_ subunit) and a phenylalanine at position 117 (N-terminus of F_1_ subunit) [2, 15, 16, 40, 42, 43]. Efficient cleavage of the F_0_ protein and virulence of the NDV strain are reliant on the presence of one or both arginines at positions 112 and 115 and/or the phenylalanine at position 117. Host ubiquitous intracellular proteases are able to cleave the F protein in the trans-Golgi membranes due to the presence of the polybasic amino acids. Upon arrival at the plasma membrane, these F proteins are already in the active state. After activation the homotrimers are transported via exocytosis to the viral surface. The C-terminal region creates the transmembrane domain which anchors the protein in the plasma membrane while the globular head containing the fusion peptide extends from the surface of the plasma membrane into the extracellular space to initiate fusion with the host cell membrane. Low virulent strains do not have multiple basic amino acids in the F protein cleavage site. Instead they have single basic amino acids and a leucine at position 117. Due to these differences the F proteins are not cleaved at the trans-Golgi membranes like they are in vNDV and mNDV strains. The F proteins remain in the inactive state when they reach the plasma membrane.

Synthesis of the F protein occurs along the ER as an inactive F_0_ precursor [42, 44]. To activate the F protein, F_0_ must be cleaved to functional F_2_ and F_1_  polypeptides to enable infectivity of progeny virions. These polypeptides must remain bound to the viral surface by disulfide bonds enabling the virus particles to be infectious. The two hydrophobic regions of the F_1_ polypeptide are the N-terminal fusion peptide and the transmembrane domain. The F_1_ polypeptide also contains two heptad hydrophobic repeat regions designated HRA and HRB.

Upon initial translation the F protein folds into a metastable form prior to fusion [42, 44]. Large scale conformational changes occur once fusion is activated. These conformational changes progress down an energy gradient to form a stable postfusion conformation. Active F_1_ polypeptide mediates fusion between the viral lipid membrane and host cellular membrane. Membrane fusion allows the viral genome to enter the host cell where initiation of viral replication occurs.

The second surface glycoprotein is a type II integral membrane, protein. The HN protein has a transmembrane region which, unlike the F protein, is located at the amino-terminal region of the protein [42, 44]. A hydrophobic region about 25 amino acids in length anchors the protein in the viral membrane and acts as a signal sequence. The HN protein promotes fusion of the viral and host cell membranes through interaction with the F protein [45, 46]. It is able to hemagglutinate cells by binding to sialic acid (SA) receptors [42, 44, 45]. The neuraminidase of the HN protein can cleave SA structures for viral release after replication. The HN protein has also been shown to play a role in tissue tropism independent of the amino acid sequence of the F protein [45].

**Figure 1:**
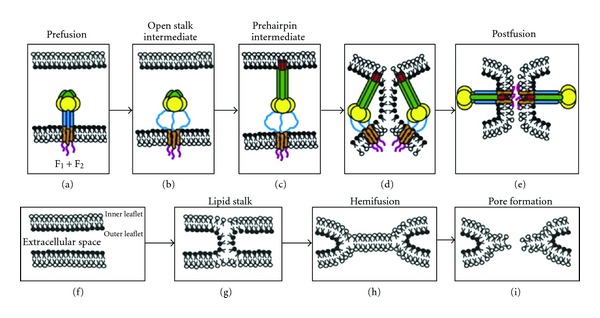
(a) The prefusion form of F contains a globular head with the HRA region in 11 distinct sections, and the HRB region is in a three-helix bundle. The F TM domain is also represented as a three-helix bundle, consistent with the oxidative cross-linking data. (b) Upon HN binding to target cells (HN not shown for clarity), F is activated for fusion, and the HRB region separates, forming the open-stalk conformation where N-1 peptide can bind to HRB. At this open-stalk stage, the TM domain is still thought to be in a three-helix bundle because N-1-HAt can still bind to HRB after the addition of the oxidative cross-linker. (c) After formation of the open-stalk conformation, HRA rearranges to form the extended *α*-helical bundle, and the FP is inserted into the target cell membrane (the prehairpin intermediate). (d-e) Finally, the postfusion state occurs with the formation of the 6-HB. (d-e and f–i) Lipid intermediates in fusion with the F protein, removed for clarity. The two bilayers contain an inner and outer leaflets and are separated by the extracellular space. During the process of F refolding to form the postfusion form, water is excluded from the extracellular space and the outer leaflets initially merge to form the lipid stalk intermediate. The lipids of the bilayers mix, forming the hemifusion intermediate, and then the fusion pore forms. F domains: FP (red), HRA (green), globular head (yellow), HRB (blue), TM domain (orange), cytoplasmic tail (pink). (From Bissonnette et al., 2009 [44] with permission.)

Replication of NDV begins by attachment of the virus to the host cell membrane. The HN protein binds to the SA receptors on the surface of the cell membrane bringing the F protein closer to the host cell [42, 44, 45]. The HN interaction with SA receptors is thought to initiate the conformational changes needed to activate the F protein.  Figure 1 is a model provided by Bissonnette et al., describing membrane fusion events [44]. During fusion events the F_1_ polypeptide undergoes additional conformational changes which expose the HRA and HRB regions [42, 44]. The two hydrophobic regions of the F_1_ polypeptide act to bind the viral membrane to the host cell membrane. The N-terminal fusion peptide attaches to the host cell membrane, while the transmembrane domain anchors the viral membrane. A 6-helix bundle (6HB) couples the free energy released during protein refolding when the two membranes merge. The final conformational state of the F protein is the most stable form and is not reversible [44].

Membrane fusion occurs at neutral pH, but the exact mechanism of fusion activation is unknown [38, 39, 42, 47, 48]. The accepted steps during the fusion event begin with docking of the viral membrane to the host cellular membrane. This docking event occurs through interaction between the HN protein and SA receptors. The F protein is activated as the membranes approach, and upon membrane merging a pore is formed between the membranes [38, 39, 43, 48–50].

The F protein alone is not sufficient for membrane fusion to occur [38, 39, 43, 46, 48–50]. Coexpression of the HN attachment protein was originally thought to be required to promote fusion. Attachment of the HN protein to the SA receptors may not be necessary to initiate activation of the F protein. NDV isolates that have mutations in the attachment function of the HN protein have been shown to continue to promote fusion. It has also been shown that lack of co-expression of the HN protein allows for fusion events to occur with some F proteins. The attachment event itself may not support the activation of the fusion process. NDV HN mutants have been shown to be unable to promote fusion while the attachment function remains intact. The attachment protein must be from the same virus as the fusion protein in order for fusion events to occur [38, 39, 43, 48–50].

The NP, P, and L proteins also play a role in replication and infectivity. All three proteins are required for viral synthesis [15–18]. The NP protein serves as the site for viral RNA synthesis and captures the genomic RNA into the nucleocapsid during replication to protect it from degradation. The concentration of free NP protein within the cell plays a role in restricting the rate of transcription and replication. The P and L proteins are nucleocapsid-associated proteins and are needed for polymerase activity. The P protein is directly involved in nascent chain assembly and binds the L protein to the NP-bound template RNA to form the RNP complex. Viral replication relies on all three proteins to produce infective virus particles.

## 7. Viral Replication

Fusion events result in pore formation allowing the viral nucleocapsid complex to enter the host cell [2, 38, 43, 48–50]. All viral replication events occur within the host cell cytoplasm. Because the genome is negative-sense RNA, the RNA-dependent RNA polymerase (L) is required to enter the cell with the genomic RNA in order for transcription to occur. Positive-sense RNA intermediates are formed which act as mRNA using the host cell translation machinery to translate proteins. Viral proteins are transported to the cell membrane for virion formation. The host cell membrane becomes modified to form the new viral envelope. The nucleocapsid proteins align within the new membrane to form the RNP complex. The new virus particles are released by budding through the host cell membrane. During viral assembly viral glycoproteins may be expressed on the surface of the host cell membrane. Accumulation of the F protein and HN attachment protein on surface of infected cells initiates fusion between neighboring cells to form syncytia [43, 44, 48]. The F protein has been shown to be capable of initiating syncytia formation without the aid of the HN protein [44, 48].

## 8. Transmission

The primary route of transmission is either by ingestion of fecal contaminated material or inhalation of droplets containing the organism [1–3, 24, 51]. Viral replication in the respiratory tract of infected birds allows for dissemination of the virus during nasal discharge. When the virus reaches the mucous membranes of susceptible birds, the virus is likely to reach the upper respiratory tract. Replication in the respiratory tract of newly infected birds allows for the potential to expose more susceptible birds and the virus easily spreads through the flock. The success of this mode of transmission hinges on the environment temperature and humidity and the viral load contained in the aerosolized droplets. Outbreaks in England from 1970 to 1971 and Northern Ireland in 1973 were attributed to respiratory inhalation of contaminated droplets. The virus is also able to replicate in the intestinal tract which can then be excreted in the feces. It has been shown that large amounts of virus are commonly excreted in the feces of NDV-infected birds.

Several methods of virus transmission have been linked to the introduction of NDV to new premises. Direct ingestion of feed or water contaminated with feces delivers a high virus load to susceptible birds [1, 2, 24]. This was demonstrated by the PPMV-1 transmission to chickens that occurred in Great Britain in 1984. Importation of sick pet or exotic birds, movement of commercial poultry and game birds or the sport of racing pigeons allows for dissemination of the virus across vast distances. A broad range of animals including reptiles and humans can be infected with NDV and are able to distribute the virus to other vulnerable animals. The virus particles have been shown to enter the eggshell after it has been laid which gives rise to the potential for virus spread during transport of table or hatching eggs. Live or attenuated vaccines may also be a source of infection if the virus used to prepare the vaccine is not properly killed or the vaccine is contaminated. Vaccination and insemination crews as well as veterinarians have been shown to transmit the disease from farm to farm due to improper cleaning and disinfecting of equipment.

Live bird markets can also contribute to the persistence and spread of the virus. These markets may not follow appropriate cleaning and disinfecting techniques which allows for the possibility of environmental contamination. Live birds in the market are exposed to birds from multiple sources. These birds run the risk of disseminating the virus as they leave the market. Low virulent strains of NDV have been regularly isolated from wild birds by the NVSL [52]. Migratory wild birds have been shown to transmit NDV to free range poultry through direct contact or by contamination of feed or water [1, 2]. In 1997, eleven outbreaks of NDV in poultry in Great Britain were linked to movement of wild birds. Double-crested cormorants have also been blamed for the spread of cormorant vND to new premises during migration. The virus is able to persist in adult cormorants as the natural reservoir allowing epidemics to occur in fledgling cormorants which are highly susceptible to this strain of NDV [2, 33].

Biosecurity of commercial poultry facilities is an important step in preventing transmission of NDV and large economic loss. It is recommended that poultry farms and hatcheries should not be in close proximity to each other to protect highly susceptible young birds [1]. Poultry farms and flock houses should also be spread apart from each other to avoid transfer of contaminated material between premises. Movement of equipment and materials between farms should be restricted and subject to thorough cleaning and disinfecting. Humans may also harbor the virus in the conjunctival sac resulting in conjunctivitis and possible dissemination of the virus [1, 24, 51]. It is not advised for people to move between premises unless appropriate biosecurity procedures are followed. Separation of farms based on species is important to prevent introduction of exotic diseases to new avian species. The water supply should be clean and should not come from surface water where migratory birds have the potential to contaminate the water source.

## 9. Pathogenesis

The pathogenicity of the virus depends on multiple factors including host species, age, immune status, secondary infections, stress, environmental conditions, the amount of virus transmitted, and the route of transmission but most importantly the strain of the infecting virus [1, 2]. Chickens are more susceptible than other species, while ducks tend to show no clinical symptoms; thus, waterfowl are considered a natural reservoir for NDV. Cleavage of the F protein during viral replication in the host plays a major role in the virulence of the virus [1, 2, 40, 48, 53]. Velogenic and mesogenic strains of NDV are able to replicate systemically due to the active state of the F protein. Unfortunately vNDV and mNDV, strains cannot be differentiated based on their amino acid sequences at the F protein cleavage site. Due to the lack of multiple basic amino acids in low virulent strains, the F protein must be cleaved by secretory trypsin-like proteases which are limited to the mucosal membranes in the respiratory and gastrointestinal tracts. Low virulent strains are not able to replicate systemically due to the limited availability of these trypsin-like proteases. Examples of vNDV, mNDV and LoNDV cleavage site sequences are shown in Table 1. 

The length of the HN protein has been shown to influence pathogenicity as well [13, 41]. The HN_0_ precursor protein is composed of 616 amino acid residues in avirulent strains of NDV including Ulster and D26 [41]. This inactive HN_0_ is converted to an active protein by proteolytic cleavage of a few nucleotides at the C-terminus. The open reading frame of other NDV strains includes stop codons upstream resulting in active proteins of 571 and 577 amino acids in length. Shortening of the HN active protein plays some role in virulence but is not completely understood.

Upon infection with NDV, macrophages of the immune system of chickens produce type I and type II interferon (IFN) [3]. Ten genes encode chicken type I IFN (ChIFN1) while only one gene is responsible for chicken type II IFN (ChIFN2). NDV is able to replicate in these macrophages despite the immune system response. Peripheral blood lymphocytes and heterophils induce apoptosis when infected with the virus. Macrophages of the respiratory system of turkeys infected with NDV show reduction in phagocytic and bacteriocidal abilities [3]. Natural immune stimulation in poultry may not be sufficient to control the disease depending on the infecting strain. Control strategies are needed to prevent development of severe disease.

## 10. Clinical Signs

The incubation period from the time of infection to development of disease varies from 2 to 15 days depending on several factors [2, 12]. The pathogenicity of the virus, host species and age, host immune status, secondary infections, stress, environmental conditions, the amount of virus transmitted, and the route of transmission can all play a role in determining the severity of disease and the length of incubation. Disease severity has led to classification of NDV isolates under three distinct pathotypes [1–3, 12, 53–55]. Infection of lentogenic NDV isolates can range from nonapparent to mild respiratory or gastrointestinal disease in adult chickens. When replication is limited to the gastrointestinal tract, the infection is often classified as asymptomatic enteric due to lack of respiratory symptoms. Young susceptible birds may develop a more serious respiratory disease that can lead to death due to increased susceptibility to secondary infection. LoNDV are categorized as lentogenic NDV and are commonly used as sources for vaccine production. Mesogenic (mNDV) isolates are considered of intermediate virulence. Infection is typically systemic and can lead to development of a nonfatal respiratory disease. Drop in egg production can be seen in layers infected with mNDV. Rarely symptoms of the nervous system can develop, but mortality is usually low following infection. Pigeon paramyxovirus isolates usually fall in the mNDV classification due to their intermediate virulence and neurologic symptoms.

Highly virulent velogenic (vND) viruses are also systemic and can cause high morbidity and mortality. Factors such as species of the infected bird, age, coinfection with other organisms, route of exposure, viral dose, stress, and the immune status of the individual determine disease severity [2, 12]. Velogenic viscerotropic ND (vvND) causes acute infection of the gastrointestinal mucosa resulting in hemorrhagic lesions and death [1, 2, 12, 30]. Clinical signs may begin with weakness, increased rate of breathing, listlessness, and prostration. During course of infection, green diarrhea, muscular tremors, and paralysis of the extremities may be apparent. Edema may be seen on the head especially around the eyes. In highly susceptible flocks, mortality can be as high as 100%.

Velogenic neurotropic NDV (vnNDV) isolates do not replicate in the gastrointestinal mucosa like vvNDV [1, 2, 12, 30]. Infection primarily leads to respiratory distress followed by neurologic disease. Drop in egg production is also seen with this strain of vNDV. Morbidity is similar to vvND, around 100%, but the mortality rate is lower. Mortality in adult birds is usually only 50%, but in young chickens it can be as high as 90%. Cormorant vNDV falls within the vnNDV classification due to the severe neurologic symptoms and high mortality rate in juvenile cormorants [3]. Clinical symptoms in turkeys may be less severe than those seen in chickens. Game birds are also susceptible, and outbreaks occasionally occur in these species. Ratites are less susceptible to disease development, while waterfowl are usually resistant [1, 2, 12].

## 11. Vaccination

Vaccination for NDV originated with the use of inactivated infective strains which were shown to provide protection in chickens [2, 3]. Inability to produce safe and effective vaccines resulted in discontinuation of large-scale production. The ability to attenuate vNDV, developed by Iyer and Dobson in 1930, enabled mNDV vaccine development [2]. Inactivated vaccines were also relied upon in the US when NDV was first introduced in the 1930's [2, 3]. Inactivated vaccines involved adsorbing the virus to aluminum hydroxide. These types of vaccines were commonly used in Europe until the third panzootic in the 1970's. The performance of inactivated vaccines was not sufficient during that panzootic so vaccination programs implemented the use of live vaccines. Mesogenic Roakin and milder Hitchner B1 and LaSota strains were developed into live virus vaccines and continue to be used today to produce live and inactivated vaccines [1, 2, 30]. Modern inactivated vaccines utilize oil emulsions instead of aluminum hydroxide resulting in more successful vaccines. Oil emulsions act as adjuvants to stimulate the inflammatory immune response [39]. Inactivated vaccines lacking an adjuvant will not induce the early immune response needed to stimulate antibody production. The adjuvant is needed to present the antigen to the immune system, localize the antigen to the inoculation site, or directly stimulate the innate immune response. Aluminum hydroxide deposits the antigen at the site of inoculation, while oil emulsions stimulate the immune response directly.

Vaccination for NDV is practiced widely in the US, and like other countries vaccine production is tightly controlled [2, 6]. OIE guidelines for vaccine production specify that live and inactivated virus vaccines must be tested extensively [2, 30]. The master seeds of live virus vaccines must have an ICPI value less than 0.4 if no less than 10^7^ 50% mean egg infectious dose (EID_50_) is inoculated in each bird or less than 0.5 if no less than 10^8^ EID_50_ are inoculated in each bird. Similarly the master seeds of inactivated viruses must have an ICPI value less than 0.7 if no less than 10^8^ EID_50_ are inoculated in each bird.

Live virus vaccines may be divided into lentogenic and mesogenic groups [1, 2, 30]. The immune response has been shown to increase as the pathogenicity of the live virus vaccine increases. To provide the best protection vaccine programs have adopted the method of progressive vaccinations which involves successive booster vaccines with increasingly virulent strains [1, 2, 30]. Another method begins with low virulent live virus vaccination followed by successive vaccinations using more virulent inactivated viruses [1, 2, 30]. This method of combining inactivated and live virus vaccines leads to stimulation of the cell-mediated, innate, and humoral immune responses to improve protection. Live virus vaccines are usually lyophilized allantoic fluid produced by infecting embryonating chicken eggs. The advantages of live vaccines include ease of administration, inexpensive production, and ease of application. Live virus stimulates a cell-mediated immune reaction which results in rapid protection after vaccination. Live viruses are able to transmit between birds so protection can be spread easily among a flock. This also results in disadvantages due to the potential for live virus vaccines to produce clinical symptoms in the flock which are again easily transmitted. Maternal antibodies can prevent live virus vaccines from immunizing young birds. Cell-mediated immune response initiated by infection by live virus does not offer complete protection against challenge. This offers an additional disadvantage for live virus vaccines.

Lentogenic live virus vaccines are administered by intranasal inoculation, eye drop, or beak dipping [2, 3, 30, 51]. Administration of mesogenic live virus vaccines is more labor intensive and includes wing-web stabbing or intramuscular inoculation. Addition of controlled concentrations of vaccines to drinking water is a popular method of vaccination. Sprays and aerosols are also a popular method for vaccine application, but the size of aerosol particles must be controlled to allow for proper inhalation. This method is usually reserved for secondary doses of vaccines to avoid severe reactions. Suboptimal vaccinations can result in “rolling reactions” where ND may cause disease that spreads between the birds of a flock resulting in increasing respiratory disease [6]. It is also possible that introduction of LoND from wild birds into a suboptimally vaccinated flock may result in the same “rolling reaction.”

Inactivated vaccines are produced using the same method as live virus vaccines, but the virus in the allantoic fluid is inactivated using beta-propiolactone or formalin [2, 3, 30]. An adjuvant (originally aluminum hydroxide and now oil emulsion) is added to the inactivated virus to stimulate the immune system. Several viruses are currently used to produce inactivated vaccines including Ulster 2C, Hitchner B1, LaSota, Queensland/V4, F, and Roakin [2, 3, 20, 30]. Administration of inactivated vaccines is limited to intramuscular or subcutaneous injection. Storage of inactivated vaccines is easier than live virus vaccines since the viability of the virus does not have to be maintained. It is labor intensive to produce inactivated vaccines due to the steps required for inactivation and testing to ensure inactivation was complete. Oil-emulsion inactivated vaccines can be used in day-old chicks because the maternal antibodies do not affect the vaccine efficiency. There is a 42-day withdrawal period between vaccination and slaughter for human consumption in the US that poses a problem for broiler chickens due to their short lifespan. No matter which type of vaccine is used, birds are still able to become infected by NDV and can transmit the disease to others [2, 5, 6]. Because vaccination cannot prevent disease transmission, its role is limited to safeguarding the individual bird from significant disease by providing protective antibodies that can quickly respond to the introduction of an ND virus [2, 5, 6].

The F and HN surface glycoproteins can elicit a protective humoral immune response [3, 5, 30, 39, 51]. Many researchers are using recombinant viruses to express these proteins for vaccine production. Fowlpox, vaccinia, pigeon pox, Marek's disease virus, retrovirus, and baculovirus have all been used as recombinant vectors to express the F and HN proteins [3]. A recombinant herpes virus expressing the F and HN proteins has been successful in protecting turkeys. Sakaguchi et al. expressed the F protein of lentogenic D26 using a recombinant Marek's disease virus [5, 56]. Mori et al. used a recombinant baculovirus to express the F protein of D26, and Lee et al. used a recombinant baculovirus to express both the F and HN proteins of LaSota and vvNDV Kr-005/00 [5, 57]. These subunit marker vaccines can provide effective antibody production while lending the ability to distinguish between natural NDV infection and vaccination [5, 20]. Research is emerging on the development of genotype and antigenically matched vaccines which are meant to eliminate viral shedding upon infection with a strain of the same genotype or antigenic characteristics [6]. Naked DNA plasmids are also being developed to express the F protein for vaccination [3].

Vaccination may cause selective pressure leading to the appearance of new strains of NDV [6, 23]. Mexico and Republic of Korea are experiencing the effects of selective pressure or ineffective vaccination. Both countries have continual outbreaks of vND in backyard flocks and have well-vaccinated birds with high levels of protective antibodies that develop a drop in egg production with the absence of clinical symptoms. Vaccination against PPMV-1 in racing pigeons is a common practice in many countries. Exposure to unvaccinated feral pigeons opens the possibility of disease transmission.

## 12. Diagnosis/Control

### 12.1. Signs/Symptoms

Diagnosis of disease begins with evaluation of clinical signs and symptoms. In chickens symptoms indicative of vND include prostration, ruffling of feathers, depression, leg and wing paralysis, or other neurologic signs along with high mortality reaching 100% in fully susceptible flocks [1, 2, 12]. Clinical symptoms in the field may not be a reliable measure of the virulence of the virus. Laboratory diagnosis is necessary for confirmation and pathotyping of NDV to rule out other diseases which may cause similar symptoms including highly pathogenic avian influenza virus.

### 12.2. Pathology

As previously mentioned the strain and the route of infection play a large role in development of clinical symptoms and lesions. Infection with panzootic vvND is commonly associated with necrosis of the intestinal wall or lymphoid tissues resulting in hemorrhagic lesions in the mucosa of the proventriculus, ceca, duodenum, jejunum, and ileum [1, 2, 12, 51]. Birds displaying neurologic symptoms do not have pathologic lesions in the central nervous system. Gross lesions of the respiratory tract may include hemorrhage of the respiratory mucosa, airsacculitis and congestion of the trachea but are not always seen. Secondary bacterial infection is a significant concern and may lead to thickened air sacs with catarrhal or caseous exudates. Infection in other organs may be marked by hemorrhage in the lower conjunctiva, paratracheal edema, and necrosis of the spleen. Laying poultry infected with vND may demonstrate flaccid and degenerative ovarian follicles, hemorrhage of reproductive organs including the ovarian follicles and egg yolk in the abdominal cavity [1, 2, 12].

Examination by histopathology also yields a variety of descriptive lesions influenced by the virulence of the strain and route of introduction. Microscopic lesions may include cellular infiltration, oedema, hyperaemia, and necrosis. Neurologic lesions are comprised of encephalomyelitis with degeneration of the neurons, lymphocyte infiltration, and hypertrophic endothelial cells. These lesions are usually found in the cerebellum, midbrain, spinal cord, medulla, and brain stem. Complete loss of cilia in the respiratory tract can occur within days of infection. In the early stages of infection, lymphocyte and macrophage infiltration is common in the mucosa of the upper respiratory tract along with congestion and edema.

Virulent strains can cause hemorrhages of the blood vessels in multiple organs especially the intestinal tract. The serosal and mucosal surfaces show marked necrosis in intestinal lymphoid aggregates. Necrosis can be seen in the cecal tonsils, and hyperplasia of monocytes is evident in the liver and other organs. The germinal centers of the spleen and thymus show marked focal vacuolation and lymphocyte destruction. Hemorrhages can also occur in the heart, gallbladder, skin, and eyelids leading to conjunctivitis. Petechiae of the wattle and combs and facial edema are commonly seen during infection. Diagnosis should not be based on pathognomonic lesions or clinical signs because these types of symptoms and lesions are not specific to any strain of NDV. Some lesions may be seen with infection of low virulent strains, and symptoms may be similar to those seen with more virulent strains. Pathology is a useful tool to guide disease diagnosis, but it cannot be used solely to diagnose ND considering these types of lesions are not unique to NDV infection.

### 12.3. Serologic Techniques

Detection of antibody is primarily used to evaluate the immune response to past infection or vaccination [1, 2, 12, 30, 51]. Generally a higher antibody titer will be seen following a more recent infection. Several diagnostic tests are available including virus neutralization in chick embryos, plaque neutralization, hemagglutination-inhibition (HI), single radial immunodiffusion, agar gel immunodiffusion (AGID), and enzyme-linked immunosorbent assay (ELISA). The ELISA and HI tests are capable of measuring titers. The ELISA consists of a microtiter plate that has NDV antigen attached to the bottom of each well. Addition of serum containing anti-NDV antibodies creates antigen-antibody binding which is detected using antibodies produced in another species against chicken antibodies. An enzyme is conjugated to the anti-chicken antibodies so when anti-NDV antibodies are present and bound to the NDV antigen, the enzyme bound to the anti-chicken antibodies catalyzes a color change in the well. This can be read by viewing the plate or quantitatively using a spectrophotometer. Serial dilution of the anti-NDV antibody test serum can be used to determine the titer.

The HI test is also performed in a microtiter plate [12, 30, 58]. The OIE standard HI method employs a V-bottom microtiter plate in which serum test specimens are serially diluted in twofold dilutions using phosphate buffered saline (PBS). A known quantity of NDV antigen (usually 4 Hemagglutinating Units) is added to each well and incubated to allow antigen-antibody binding. A 1% suspension of red blood cells (RBCs) is added to each well and incubated again. The hemagglutinin protein on the envelope of NDV binds RBC's resulting in what is referred to as hemagglutination. Unbound antigen in the HI test is able to hemagglutinate the RBC's in the absence of anti-NDV antibody resulting in a diffuse red color throughout the well. In the presence of anti-NDV antibody, the antigen is not allowed to hemagglutinate the RBC's because the hemagglutinin protein is bound to and blocked by the anti-NDV antibody. The RBC's settle into a distinct pellet on the bottom of the well, and tilting the plate at a 45° angle will result in a teardrop pattern in wells where the antigen is fully inhibited. The teardrop pattern of each serum sample should be compared to that of a known antibody control diluted using the same method described previously.

The NVSL employs a slightly different version of the HI test method. U-bottom microtiter plates are used instead of V-bottom plates [59]. The NDV antigen is added to the plate, and the serum is diluted directly in the antigen leaving out the need for PBS in the test wells. The RBC's are prepared in a 0.5% suspension instead of the 1% suspension used in the standard method. The serum HI titer for both methods is determined by taking the reciprocal of the highest dilution of test serum which is able to completely inhibit hemagglutination of the RBC's [1, 2, 12, 30, 58, 59]. Test serum may cause nonspecific agglutination of RBC's so adsorption with chicken RBC's to remove serum agglutinins should be done on serum prior to testing.

### 12.4. Virus Isolation

 Virus can usually be isolated from tracheal/oropharyngeal swabs, fecal or cloacal swabs from live birds, or tissues collected from affected organs of dead birds [1, 2, 12, 51, 60]. Intestinal tissue and trachea are the most likely organs to contain virus, but other organs demonstrating clinical signs could be used for virus isolation. PPMV-1 replicates in the brain causing neurologic symptoms. Brain tissue may be used for isolation, but it is not recommended to pool the brain with any other tissue. It is also not recommended to pool tracheal and fecal tissues [60]. Swabs are collected in viral transport media such as brain heart infusion (BHI) broth. The swab is swirled to release the viral particles into the media then wrung out along the inside of the tube so the swab can be removed prior to transport to the lab. Removal of the swab prevents the media from being absorbed to allow for more media available for virus isolation and molecular testing.

Upon arrival in the laboratory, tissues are homogenized to a 20% weight/volume suspension in antibiotic media such as BHI broth. Swab media and tissue homogenates are centrifuged to separate the heavier elements from the viral particles in the supernatant. A portion of the swab supernatant is added to an antibiotic mixture and incubated for at least one hour to eliminate bacterial contamination. The swab or tissue suspension is then used to inoculate a culture system such as chicken embryo kidney (CEK) cells, chicken embryo fibroblast (CEF) cells, or specific-pathogen-free (SPF) embryonating chicken eggs [1, 2, 12, 60]. The SPF chicken egg is the most commonly used culture system.

When SPF eggs are not available, eggs can be used from flocks that do not have antibodies to NDV. Eggs are incubated 9–11 days at 37°C prior to inoculation. Four to five eggs are inoculated into the allantoic cavity with 0.2 mL to 0.3 mL of the antibiotic-treated suspension and incubated at least four days at 37°C in a humid incubator. Inoculated eggs are examined daily for embryo mortality. The allantoic/amniotic fluid (AAF) is harvested from dead embryos on the same day they die to reduce hemolysis of RBC's within the egg. At the end of the incubation period, live embryos are chilled at 4°C to kill the embryos and the AAF is harvested.

Presence of live virus in the AAF is determined by the hemagglutination (HA) test. As previously described, the hemagglutinin surface glycoprotein of NDV binds RBC's resulting in hemagglutination. In the HA test PBS is added to all wells of a microtiter plate and the harvested AAF is serially diluted twofold across the plate. RBC's are added and allowed to incubate approximately 30 minutes, and the plate is tilted to evaluate the wells for the presence or absence of a teardrop pattern. Wells with a teardrop formation do not contain any or enough viral antigen to agglutinate the RBC's. Wells exhibiting hemagglutination have a diffuse red color throughout the well. This red color is the result of the agglutination of RBC's and antigen forming a lattice.

Specimens which are not positive for hemagglutinating virus should be passaged through embryos at least one more time. Cormorant vNDV does not always demonstrate the ability to hemagglutinate RBC's. Replacement of chicken RBC's with turkey RBC's may be beneficial when testing viral-infected AAF isolated from cormorant species. Even when turkey RBC's are utilized, the HA activity of the virus remains low making the HA test unreliable for evaluating this strain of NDV. Bacteria may cause hemagglutination leading to a false-positive result. Contamination of AAF should be evaluated using a culture method such as 24-hour incubation on a blood agar plate. Contaminated AAF can be filtered through a 450 nm membrane and passaged again in embryos.

Hemagglutinating virus can be evaluated by the HI test specific for APMV-1 [1–3, 12, 61]. U-bottom microtiter plates are set up using a method similar to the one previously described for serum specimens. The unknown AAF is diluted to 4 HA units and added to each well in one row of the plate. A known reference antigen is also added to the wells of one row. APMV-1-positive polyclonal antibody diluted to a known concentration is serially diluted twofold in the AAF across the row. The AAF and antibody are allowed to incubate for 30 minutes then a 0.5% suspension of RBC's is added to each well. Following an additional 20-minute incubation, the plate is tilted at a 45° angle and evaluated for tear-drop formation using the same method described previously. Monoclonal antibodies (mAb) can also be used in the HI test to identify antigenic groups including PPMV-1, mAb B79 which reacts with almost all NDV except Class I isolates, other lentogenic mAb (such as AVS-1 and 15C4), and vNDV (mAb 10D11C) [2, 3, 6, 12, 26, 62]. Additional characterization is needed to assess the virulence of the isolate in order to develop control measures during an outbreak.

### 12.5. Characterization

Historically three *in vivo *methods have been used to determine pathogenicity [2, 3, 12, 30, 63, 64]. These methods include (1) mean death time (MDT) in embryonating chicken eggs, (2) intravenous pathogenicity index (IVPI), and (3) ICPI. When performing the MDT procedure, tenfold serial dilutions of clean AAF are prepared and each dilution is inoculated into five 9-to-11-day-old embryonating chicken eggs via the allantoic sac route [65]. The inoculation time is recorded. A second group is inoculated in the same manner as the first, and the inoculation time is again recorded. The eggs are incubated at 37°C and candled twice a day, once at the beginning of the day and once at the day's end. Candling and incubation continues until all embryos die which may require up to seven days. The minimum lethal dose (MLD) is considered to be the highest dilution that killed all 5 embryos. The time in hours for all five embryos to die for each set of the MLD is averaged, giving the MDT.

The IVPI test requires bacteria-free viral-infected AAF with an HA titer greater than 1 : 16 [12, 30, 64]. The AAF is diluted 1 : 10 in sterile saline, and 0.1 mL is intravenously inoculated into 10 six-week-old SPF chickens. Birds are examined daily for a 10-day period, and each bird is scored according to the following observations: 0 if the bird is normal, 1 if the bird demonstrates signs of sickness, 2 if the bird is severely ill, and 3 if the bird is dead. If a bird is dead, it must be recorded as 3 for each observation for the remainder of the 10-day period. The IVPI is calculated as the mean of each observation for each individual bird over the 10 day period. The index can range from 0.00 meaning no birds became ill or died over the observation period to 3.00 meaning the virus killed all 10 birds in the first 24 hours after inoculation. ICPI is the accepted *in vivo* method of determining pathogenicity for NDV according to OIE standards [1–3, 12, 30]. As described previously an ICPI greater than or equal to 0.7 is one of the virulence criteria requiring reporting of NDV to the OIE. Table 1 shows ICPI, MDT, and IVPI pathogenicity indices for 9 well-characterized ND viruses.

Any ICPI value above 0.7 is classified in the mesogenic to virulent range [2, 3]. Virulent isolates usually vary from 1.54 to 1.9 (maximum value is 2.0). Cormorant vNDV and PPMV-1 or vNDV isolates recovered from psittacine birds can have a highly variable range of ICPI values [2, 3, 6, 26]. These isolates have been shown to have ICPI values from 0.69 to 1.45. These values place them in the mesogenic range which classifies them as select agents. Additional passages of these strains of NDV through embryonating chicken eggs may lead to increased ICPI values indicating adaptation to poultry over time. Viral tropism for PPMV-1 during chicken inoculation studies include the heart and brain which is normally not seen in infected psittacines or from inoculation with other strains of vNDV [26]. For these reasons psittacine origin NDV such as PPMV-1 transmission to poultry continues to be highly concerning.

Velogenic strains of NDV can be classified as either velogenic viscerotropic or velogenic neurotropic based on the results of the intracloacal inoculation test [3, 28, 63, 66]. The intracloacal inoculation test also requires bacteria-free viral-infected AAF diluted 1 : 10 in sterile saline. The cloaca of four six-to-eight-week-old SPF chickens are swabbed with the diluted inoculum. Birds are examined daily for a 10-day period. All dead birds are necropsied and scored according to the following observations: +4 when edema of the head and neck, hemorrhage in the trachea, and hemorrhage and necrosis throughout the gastrointestinal tract are present and +3 to +1 when lesions exist in the respiratory and intestinal tracts but have decreased severity. Viruses are determined to be vvNDV if one bird has +4 lesions or at least two birds have +2 to +3 lesions. When birds display neurologic signs prior to death, the virus is classified as vnNDV.

 The need for trypsin in cell culture media has also been used as an indication of pathogenicity [2, 3, 6, 23, 30]. As previously described, in order for the F protein to become active, it must be cleaved by secretory trypsin-like proteases. These types of proteases are limited to the mucosal membranes in the respiratory and gastrointestinal tracts. Low virulent strains are not able to replicate systemically due to the limited availability of these trypsin-like proteases. Virulent strains of NDV are able to replicate systemically due to the presence of multiple basic amino acids at the Fusion protein cleavage site which make it easier to cleave by non-trypsin-like proteases. This is also true for *in vitro* analysis where vNDV is able to replicate and cause plaques in cell culture system lacking trypsin-like proteases. Low virulent strains are limited in the cell culture system in which they are able to replicate due to the lack of trypsin-like proteases. All NDV isolates are able to replicate in CEK cells likely due to the presence of trypsin-like proteases. Cell culture systems like CEF and mammalian cell lines must be supplemented with trypsin-like proteases or exogenous proteases provided by allantoic fluid, in order for LoNDV to replicate.

### 12.6. rRT-PCR

As described in the Section 12.4, classical diagnostic techniques such as virus isolation and chicken pathogenicity testing can be time consuming [2, 6, 24, 30, 67]. Rapid diagnostic tests such as rRT-PCR and sequencing to determine pathogenicity greatly reduce the time required for implementing control measures. Molecular diagnostic assays have come a long way from conventional polymerase chain reaction (PCR) followed by gel electrophoresis for amplicon analysis to the current methods of real-time reverse transcription (rRT) PCR using one-step PCR enzyme kits [68]. RT-PCR assays provide quick amplification helping them to become essential diagnostic tools for viral detection. The negative sense nature of the NDV RNA requires reverse transcription into complimentary DNA (cDNA) prior to RT-PCR amplification. Reverse transcription of single-stranded RNA results in single-stranded cDNA using an RNA-dependent DNA polymerase enzyme [68, 69]. Advances in enzyme technology allow both steps to be done in a single tube on a single PCR instrument. Several rRT-PCR assays have been developed to detect different genes of NDV including the Matrix, Fusion, and RNA-dependent RNA polymerase.

During rRT-PCR the reverse transcriptase enzyme becomes activated at around 45°C initiating reverse transcription of the RNA to cDNA [68–70]. The enzyme is allowed to reverse-transcribe for approximately 10 minutes. When ample cDNA has been produced, the temperature is brought up to 95°C for an additional 10 minutes which causes the reverse transcriptase to become inactivated. After enzyme inactivation the instrument enters a cycling stage which usually involves advancing through three temperature settings. The first temperature, usually 94°C, causes quick denaturing of the double-stranded cDNA into single-stranded cDNA. The second step allows for a set of nucleotide primers usually located within less than 500 bases of each other along a gene to anneal to the single-stranded cDNA. One primer binds the positive sense strand and the other binds the negative sense strand. In real-time PCR a nucleotide probe also binds to its target cDNA sequence located somewhere between the two primer sequences during this step. The temperature of the second step is determined by the melting temperature of the less stable primer and the melting temperature of the template. The third step is usually at 72°C, where the polymerase begins at the primer binding site and copies the cDNA in the 3′ to 5′ direction along each strand. This results in two sets of double-stranded cDNA from each original cDNA strand. These three steps, denaturing, annealing, and amplification, are repeated up to 40 or more times resulting in a doubling of cDNA during each stage.

When real-time PCR enters this cycling stage, the probe becomes the indicator of cDNA amplification. The TaqMan probe contains a fluorescent reporter dye, such as 6-carboxyfluorescein (FAM), on the 5′ end and a quencher, such as the black-hole quencher, located at the 3′ end [68–70]. The proximity of the quencher to the fluorescent reporter dye reduces fluorescence from being released by fluorescence resonance energy transfer (FRET) through space. The polymerase moves along the gene extending the primer toward the site where the probe, is located. When the polymerase runs into the probe the fluorescent dye is cleaved from the probe by the 5′ exonuclease activity of the *Taq* polymerase. Since the fluorescent dye is no longer in close proximity to the quencher, it is allowed to release fluorescence. The probe is subsequently cleaved from the target strand, and the polymerase copies the remainder of the cDNA strand. The PCR instrument detects the fluorescence and records the accumulation of fluorescence as the cycling stages progress. The amount of fluorescence detected is directly proportional to the amount of fluorophore released during the polymerase exonuclease activity. This is also directly proportional to the number of cDNA copies produced during each cycle which is relayed by the instrument as the cycle threshold (Ct) value. The Ct value is determined to be the number of PCR cycles at which exponential increase in cDNA copies is detected by the release of fluorescence.

Detection methods other than TaqMan probes are available and have been utilized for NDV rRT-PCR assays. Fluorescent dyes such as SYBR Green or LUX can be used in RT-PCR assays without the need for a labeled probe [71]. The LUX dye is used to label one primer with a single fluorophore near the 3′ end. A hairpin at the 3′ end acts to quench the fluorescence which is then released when the primer binds the template DNA. SYBR Green intercalates into dsDNA, and fluorescence is released as the dsDNA melts during each RT-PCR cycle. The amount of fluorescence is proportional to the concentration of DNA in the sample. Some studies have used SYBR Green in conjunction with melt curve analysis for differentiating vNDV from LoNDV [72, 73]. Melt curve analysis employs intercalating dyes such as SYBR Green or LUX fluorogenic primers to measure the change in fluorescence during each cycle which can then be plotted against the melting temperature (Tm) [71–73]. Virulent and low virulent strains have differences in GC content, sequence composition, base mismatches, and amplicon length which contribute to the Tm of each strain. The calculated melt curve can be used to differentiate between strains [72, 73]. The melt curve must also be analyzed to identify nonspecific amplification such as primer dimers.

Benefits of melt curve analysis include primer design and cost. One set of primers can be used to differentiate vNDV from LoNDV decreasing the need for multiple primers in one assay. Costs are lower because expensive fluorescent labeled probes are not employed and multiple primers do not have to be purchased. Decrease in sensitivity is a disadvantage of using intercalating dyes such as SYBR Green [71]. These dyes are not bound to a probe and therefor have the ability to intercalate non-specifically into any dsDNA present such as primer-dimers or non-target amplicons. Analyzing melting curves for individual isolates can be subjective. The Tm of individual isolates may not vary significantly and can be difficult to distinguish between low virulent and highly virulent strains [72]. TaqMan probes have been used extensively in rRT-PCR assays for detection and differentiation of NDV [4, 7, 11, 13, 29]. These fluorescent labeled probes have the advantage of binding to specific regions in the target amplicon which decreases the risk of nonspecific fluorescence. This type of detection system may require multiple primer sets and sometimes several different TaqMan probes to differentiate between LoNDV and vNDV strains which can be costly [72, 73]. Probes may also degrade during their lifespan and release non-specific fluorescence [71].

The USDA-validated rRT-PCR assays used at the NVSL and the NAHLN laboratories detect the M and F genes of NDV. Both assays utilize TaqMan probes and* Taq* polymerase enzyme. The M gene assay was designed to detect the highly conserved matrix gene of most APMV-1, mainly Class II viruses [4, 6–8]. This assay is used as a screening tool to detect APMV-1 in diagnostic samples or allantoic fluid from inoculated embryos. The M gene assay has been highly successful for APMV-1 detection including vNDV, cormorant vNDV, PPMV-1, and most LoNDV. A recent publication by Kim et al. has shown that the M gene assay failed to detect 73% of Class I isolates between 2004 and 2007 [4, 8]. Low virulent Class I isolates recovered from waterfowl and live bird markets in the US contained genomic variability in the probe binding site causing loss of probe binding. This lack of detection causes significant concern because of the possibility of one of these undetected isolates converting from low virulent to virulent. The possibility of not detecting a new strain of vNDV could lead to an outbreak and significant economic loss. Development of a new M gene assay with the ability to detect a broader range of APMV-1 could reduce the possibility of failing to diagnose a new vND outbreak.

Specimens testing positive by the M gene assay are subsequently tested by the F gene rRT-PCR assay. The F gene assay is designed to only detect virulent strains of APMV-1 by binding the F gene cleavage site [6, 7, 67]. This assay was validated during the California END outbreak in 2002-2003 at the NVSL. Since that time it has been used by the NVSL and NAHLN laboratories for vND diagnostic testing. Recent analysis by Kim et al. indicates some PPMV-1 strains are not detected by the F gene assay [6, 7]. Mismatches in the probe binding site of some strains of PPMV-1 are also to blame for the lack of detection of these viruses. Another publication by Rue et al. indicates the F gene assay is also unable to detect cormorant vNDV [11]. The inability to detect virulent strains of APMV-1 is highly concerning because of the potential economic loss an introduction of vND can cause. Even though PPMV-1 and cormorant vND are endemic in pigeons and cormorants, respectively, detection of any strain of vNDV is important in the US because either strain may have the potential to infect poultry. Development of a new F gene assay able to detect all strains of vNDV would be beneficial for vND passive surveillance efforts including diagnostic testing for avian mortality events and foreign animal disease diagnostic investigations.

### 12.7. Sequencing

DNA sequencing is used in the diagnostic laboratory to analyze the virulence potential of APMV-1 isolates. Sequencing techniques originated with Sanger et al. using the dideoxynucleotide triphosphate (ddNTP) mediated chain termination and Maxam et al. using the chemical degradation method [74–76]. Sequencing techniques have been rapidly improving since that time, and next-generation or automated sequencing techniques have become standard for laboratory analysis of gene sequences. In general sequencing occurs as follows; template-specific primers are used to amplify cDNA to a high copy number [77]. The amplified cDNA is added to a second PCR reaction where DNA polymerase extends template-specific primers by addition of single-fluorescent ddNTPs. The fluorescence of each ddNTP added during chain extension is detected using an automated sequencing instrument. The position of each nucleotide is identified according to the distance from the primer. The identity of the nucleotide in the DNA chain is identified by the individual label of each ddNTP incorporated.

According to the OIE the molecular characteristics of reportable virulent NDV include multiple basic amino acids at the carboxyl (C-) terminus of the APMV-1 F_2_ protein and the presence of phenylalanine at residue 117 of the F_1_ protein N-terminus [1–3, 7, 30]. The presence of multiple basic amino acids allows the F gene of vNDV to be easily cleaved by host ubiquitous intracellular proteases present throughout the body [1, 2, 30, 78]. The virus is allowed to replicate systemically in any host tissue leading to serious disease development. At the NVSL the presence of multiple basic amino acids is detected by sequencing a short region of the F gene encompassing the cleavage site. The resulting nucleotide sequence is converted into an amino acid sequence which is then analyzed manually to evaluate the amino acids present directly upstream of the cleavage site.

One or two pairs of basic amino acids, usually lysine (K) or arginine (R) followed directly by phenylalanine at residue 117, indicate a virulent or mesogenic sequence [6, 13, 29, 40]. Consensus sequences for virulent and mesogenic strains have been determined by Collins et al. to be ^112^R/K-R-Q-R/K-R-F^117^ [3, 13, 40, 79]. Molecular analysis at the F gene cleavage site cannot differentiate mesogenic and virulent strains. PPMV-1 isolates have been shown to have one of two consensus sequences ^112^G-R-Q-K-R-F^117^ or ^112^R-R-K-K-R-F^117^[6, 40, 79, 80]. Low virulent strains usually have a single basic amino acid followed by a leucine at residue 117. Consensus sequences for low virulent strains have been determined to be ^112^G/E-K/R-Q-G/E-R-L^117^. Examples of amino acid sequences between positions 112 and 117 of the FO cleavage site for 9 well-characterized APMV-1 strains are shown in Table 1. Low virulent strains are commonly isolated in the US from wild birds, live bird markets, and poultry vaccinated with live virus. Fusion gene sequencing analysis is important to monitor endemic low virulent strains to ensure mutations are not present which may lead to conversion to virulence.

## 13. Conclusions

APMV-1 affects all orders of avian species and has a world-wide distribution. Disease severity is dependent on several factors including route of inoculation, host species, and pathogenicity of the virus strain. Virulent strains can cause great economic loss to the agricultural community. Maintaining up-to-date diagnostic assays is important for passive surveillance including mortality events and foreign animal disease diagnostic investigations. Similarities in clinical symptoms to other avian diseases and ease of viral transmission emphasize the need for rapid diagnosis. Classical diagnostic methods such as virus isolation can be time consuming leading to a delay in viral identification and characterization. Molecular methods including rRT-PCR assays and sequencing techniques are rapid methods of identifying and pathotyping viruses based on their genetic characteristics. Genetic diversity can limit value of molecular assays if primer design does not keep up with changes in viral sequence over time.

Small genomic changes resulting from replication errors can result in alterations in virulence. Introduction of basic amino acids in the fusion gene cleavage site, for example, can grant the virus ability to replicate systemically and cause severe disease. Molecular changes should be monitored to analyze alterations in the cleavage site which can identify a potential increase in virulence. Sequencing and rRT-PCR assays remain important diagnostic tools for monitoring viral changes. Molecular assays should be continually modified to maintain the ability to detect all strains of APMV-1.

 Differentiation between virulent and low virulent strains is also important to recognize and respond to introductions of virulent viruses into the US. RRT-PCR assays play an important role in detecting virulent strains of APMV-1. The ability to detect vND introduction in poultry remains a priority for diagnostic laboratories. The fusion gene should continue to be analyzed for development of an rRT-PCR assay with the ability to detect all strains of vNDV.

## Figures and Tables

**Table 1:** Examples of ICPI, MDT, IVPI, and cleavage site for some strains of NDV.

APMV-1 Strain	Pathotype	ICPI	MDT	IVPI	Cleavage site sequence
Ulster 2C	Asymptomatic enteric	0.0	>150	0.0	^112^G-K-Q-G-R-L^117^
Queensland V4	Asymptomatic enteric	0.0	>150	0.0	^112^G-K-Q-G-R-L^117^
Hitchner B1	Lentogenic	0.2	120	0.0	^112^G-R-Q-G-R-L^117^
NJ-LaSota	Lentogenic	0.4	103	0.0	^112^G-R-Q-G-R-L^117^
NJ-Roakin (Daubney)	Mesogenic	1.45	68	0.0	^112^R-R-Q-K-R-F^117^
Beaudette C	Mesogenic	1.6	62	1.45	^112^R-R-Q-K-R-F^117^
Texas Gilbert Boney 1948	Velogenic neurotropic	1.75	55	2.7	^112^R-R-Q-K-R-F^117^
Italian	Velogenic	1.85	50	2.8	^112^R-R-Q-R-R-F^117^
England-Herts- 33/56	Velogenic	2.0	48	2.7	^112^R-R-Q-R-R-F^117^

* Information obtained from Diseases of Poultry 12 Ed. Tables 3.2, page 81 and 3.4, page 87, Liu (2009) et al. [22], and from NVSL.
